# Tanzanian primary healthcare workers’ experiences of antibiotic prescription and understanding of antibiotic resistance in common childhood infections: a qualitative phenomenographic study

**DOI:** 10.1186/s13756-021-00952-5

**Published:** 2021-06-27

**Authors:** Matilda Emgård, Rose Mwangi, Celina Mayo, Ester Mshana, Gertrud Nkini, Rune Andersson, Sia E. Msuya, Margret Lepp, Florida Muro, Susann Skovbjerg

**Affiliations:** 1 Department of Infectious Diseases, Institute of Biomedicine, Sahlgrenska Academy, University of Gothenburg, Gothenburg, Sweden; 2 Centre for Antibiotic Resistance Research (CARe), University of Gothenburg, Gothenburg, Sweden; 3 Department of Paediatrics, Queen Silvia Children’s Hospital, Sahlgrenska University Hospital, Gothenburg, Sweden; 4 Institute of Public Health, Kilimanjaro Christian Medical University College (KCMUCo), Sokoine Road, Moshi, Tanzania; 5 Department of Community Health, Kilimanjaro Christian Medical Centre (KCMC), Moshi, Tanzania; 6 Department of Clinical Microbiology, Sahlgrenska University Hospital, Region Västra Götaland, Gothenburg, Sweden; 7 Institute of Health and Care Sciences, Sahlgrenska Academy, University of Gothenburg, Gothenburg, Sweden; 8 Østfold University College, Fredrikstad, Norway; 9 School of Nursing and Midwifery, Griffith University, Gold Coast, QLD Australia

**Keywords:** Drug prescribing, Phenomenography, Antimicrobial stewardship, Drug resistance, Bacterial

## Abstract

**Background:**

Antibiotic resistance is a threat to global child health. Primary healthcare workers play a key role in antibiotic stewardship in the community, but few studies in low-income countries have described their experiences of initiating antibiotic treatment in children. Thus, the present study aimed to describe primary healthcare workers’ experiences of antibiotic prescription for children under 5 years of age and their conceptions of antibiotic resistance in Northern Tanzania.

**Methods:**

A qualitative study involving individual in-depth interviews with 20 prescribing primary healthcare workers in Moshi urban and rural districts, Northern Tanzania, was performed in 2019. Interviews were transcribed verbatim, translated from Kiswahili into English and analysed according to the phenomenographic approach.

**Findings:**

Four conceptual themes emerged during the analysis; conceptions in relation to the prescriber, the mother and child, other healthcare actors and in relation to outcome. The healthcare workers relied mainly on clinical examination and medical history provided by the mother to determine the need for antibiotics. Confidence in giving advice concerning non-antibiotic treatment varied among the participants and expectations of antibiotic treatment were perceived to be common among the mothers. Antibiotic resistance was mainly perceived as a problem for the individual patient who was misusing the antibiotics.

**Conclusions:**

To increase rational antibiotic prescription, an awareness needs to be raised among Tanzanian primary healthcare workers of the threat of antibiotic resistance, not only to a few individuals, but to public health. Guidelines on childhood illnesses should be updated with advice concerning symptomatic treatment when antibiotics are not necessary, to support rational prescribing practices and promote trust in the clinician and mother relationship.

**Supplementary Information:**

The online version contains supplementary material available at 10.1186/s13756-021-00952-5.

## Background

The increasing antimicrobial resistance of common human pathogens has been recognised as a public health emergency by the World Health Organisation (WHO) [1]. Children, in particular neonates, in low-income countries are disproportionally affected due to higher susceptibility and severity of infectious diseases, as well as limited access to diagnostic tools and treatment [2]. All antibiotic use, whether appropriate or not, can promote the emergence of resistance in bacteria. Rational use of antibiotics, i.e. only using antibiotics when necessary and at an appropriate dosage and duration of treatment, is an important strategy to preserve antibiotic effectiveness for future generations [3].

An inverse relation between the prevalence of antibiotic resistance in invasive isolates and the income status of a country has been shown [4], revealing that low-income countries not only have a higher burden of infectious diseases but also higher prevalence of antimicrobial resistance. The global antibiotic consumption rate per capita has increased by 39% between 2000 and 2015, driven by increased consumption in low- and middle-income countries [5]. Although lack of access to effective antibiotics remains a serious challenge in some low-income settings [6], antibiotics are also widely used in children in many low-income countries, including Tanzania [7]. In Northern Tanzania, antibiotics are frequently sold and dispensed without a prescription [8, 9], although antibiotics are classified as prescription-only drugs. Furthermore, antibiotics are not only used in humans but also in animals and agriculture. For example, antibiotics are commonly used to increase growth in livestock animals by pastoralists in Tanzania [10]. Meanwhile, a study on associated factors driving global antimicrobial resistance concluded that reducing antibiotic consumption alone will not be sufficient to control antibiotic resistance as long as resistant strains and genes are spread in the environment due to poor infrastructure, including inadequate sanitation and water purification. Naturally, these factors are often aggravated in deprived, over-crowded settings [11]. This explains why low- and middle-income countries are more vulnerable to increased antibiotic resistance although antibiotic consumption has only recently reached the same levels as high-income countries [5]. Several interconnected human, animal and environmental habitats indeed contribute to the emergence and spread of antibiotic resistance which calls for a ‘One Health’ approach [12].

Knowledge, attitudes and perceptions of antibiotics and resistance have been extensively studied among dispensers and drug-shop owners in Tanzania [13–15]. These studies indicate an awareness of antibiotic resistance and knowledge of rational antibiotic use among the majority of these professionals. However, when patients have prescriptions from a clinician, they are less likely to act upon this knowledge [15]. Thus, healthcare workers’ prescribing practices may have ‘legitimised’ inappropriate dispensing and use of antibiotics among the public [16]. Previous studies in Moshi, Northern Tanzania have shown worrying signs of over-prescription by clinicians both at the hospitals and at a primary healthcare level [17, 18]. However, few studies have portrayed Tanzanian primary healthcare workers’ experiences and underlying conceptions of antibiotic use in childhood illnesses, which are of considerable importance when shaping strategies to promote antibiotic stewardship. Thus, the present study has sought to describe primary healthcare workers’ experiences of antibiotic prescription for children under 5 years of age and their understanding of antibiotic resistance in Moshi, Tanzania.

## Methods

### Study design and approach

This qualitative study was conducted using a phenomenographic approach, retrieving data from individual in-depth interviews. The aim of phenomenography, first described by Marton [19], is to describe the way in which a group of people make sense of, experiences and understand a phenomenon in the world around them. The aim of phenomenography is to take the ‘second order of perspective’, that is describing the world as experienced by people rather than making statements concerning the world itself [20]. This approach was developed through studies of learning in higher education [19], and has since been applied to describe the different ways in which healthcare professionals perceive medical practice, including antibiotic prescribing practices and conceptions of antibiotic resistance [21–24].

### Study setting

The study was conducted in Moshi Municipal (urban) and Moshi District (rural) Council, in the Kilimanjaro region of Northern Tanzania. In the 2012 census the total number of inhabitants in the Kilimanjaro region was about 1.6 million, while the approximate population in Moshi urban and rural districts was 184,000 and 467,000, respectively [25]. The Kilimanjaro region is considered to be a low malaria endemic area [26]. The primary healthcare system in Tanzania has two levels of facilities, dispensaries and health centres. The majority of these facilities are public, but some are also private or are run by a faith-based organization. Dispensaries are smaller and often less well-equipped compared to health centres which sometimes have a labour ward and are able to admit patients. However, private or faith-based dispensaries are often functioning at the level of public health centres. In Moshi urban district there are eight health centres and 47 dispensaries whilst Moshi rural district comprises eight health centres and 78 dispensaries. There are two secondary referral hospitals in Moshi urban district, Mawenzi and St. Joseph Hospital, whereas in Moshi rural district there is one, Makuyuni Hospital in Himo. Further, Kilimanjaro Christian Medical Centre (KCMC) situated in Moshi urban district, is the second-largest consultant hospital in the country serving as a tertiary referral and teaching hospital for Northern and Central Tanzania. Ninety percent of the inhabitants in Kilimanjaro region are estimated to live within 5 km of a healthcare facility [27]. In Tanzania, the government endorse a ‘no fee health service policy’ for children under the age of five and antenatal care to promote equitable healthcare [28]. Generally, Tanzanian mothers are caring for the smaller children or, at times they delegate this task to a house maid or relative, whilst fathers are expected to yield the main source of income. This is reflected at the health facilities where the vast majority bringing children to the clinic are women.

### Study sites and participants

Recruitment of healthcare workers took place in September–November 2019 at five health centres and seven dispensaries, chosen to represent different geographical and socioeconomic parts of Moshi urban and rural district. The healthcare facilities were both governmental (n = 8), private (n = 3) and faith-based (n = 1). The head of each facility was visited by a member of the team prior to data collection to inform about the aims of the study and to demonstrate approvals. All of the governmental and faith-based facilities approached agreed to participate, while one private facility declined. After receiving permission, a suitable day and time to conduct the interviews was agreed upon. All healthcare workers prescribing antibiotics were eligible for inclusion. In all dispensaries except one, only a single prescribing clinician was present. Each health centre had several prescribing clinicians on duty. In health facilities with several prescribing clinicians, 2–3 were included depending on availability and in order to represent a variety in educational backgrounds. In total, 20 healthcare workers were included in the study (see Table 1 for participant demographics).

**Table 1:** *Characteristics of the participants*

Healthcare worker	Age	Gender	Educational background	Years of experience^b^	Level	Owner	Area	District
1	31–35	Male	CO	9	Dispensary	Public	Urban	MU
2	N/A	Male	CO	5	Dispensary	Private	Rural	MR
3	51–55	Female	CO	10	Dispensary	Public	Rural	MR
4	31–35	Female	Nurse	5	Dispensary	Public	Rural	MR
5	41–45	Male	AMO	12	Health Centre	Public	Urban	MU
6	26–30	Female	CO	5	Health Centre	Public	Urban	MU
7	46–50	Male	MD	20	Health Centre	Public	Urban	MU
8	46–50	Male	CO	14	Health Centre	Private	Urban	MU
9	51–55	Male	CO	27	Health Centre	Private	Urban	MU
10	46–50	Female	CO	18	Dispensary	Public	Semi-urban	MU
11^a^	51–55	Female	AMO	15	Health Centre	Public	Urban	MU
12	31–35	Female	MD	3	Health Centre	Public	Urban	MU
13	31–35	Male	MD	2	Health Centre	Public	Urban	MU
14	36–40	Female	CO	10	Dispensary	Public	Semi-urban	MU
15	36–40	Female	CO	10	Dispensary	Public	Semi-urban	MU
16	56–60	Male	CO	33	Health Centre	Private	Urban	MU
17	61–65	Male	MD	30	Health Centre	Private	Urban	MU
18	N/A	Female	MD	3	Health Centre	Public	Rural	MR
19	N/A	Female	Nurse	13	Health Centre	Public	Rural	MR
20	N/A	Male	CO	3	Dispensary	Faith-based	Rural	MR

*N/A* no answer, *CO* clinical officer, *AMO* assistant medical officer, *MD* medical doctor, *MU* Moshi urban, *MR* Moshi rural

^a^Interview not recorded, data retrieved from notes

^b^Years of experience prescribing antibiotics

### Data collection

Prior to data collection three local research assistants (CM, EM and GN) were appointed by RM. The team was provided with training relating to the background, design and methodology of the study by ME, ML and RM. A local, senior paediatrician was consulted on medical aspects during the team training sessions. The interview guide was developed by ME, ML and RM and piloted on two medical interns employed at KCMC. Subsequently, and during the first part of the data collection, some follow-up questions were added to support the interviewee when reflecting on the main research question. The guide (in English) was translated into Kiswahili by CM, EM, GN and RM (Additional file 1). The individual interviews took place at the clinician’s private workplace and lasted between 15 and 48 min. Each interview was conducted by two members of the team (CM, EM, GN, ME or RM), one was the main interviewer while the other handled audio recording and took notes for backup. All interviews were held in Kiswahili. The interview started with the main interviewer giving verbal information about the study, followed by some standard questions about each participant’s demographic and educational background. Each interview opened with the following question *“Can you please describe your experiences of antibiotic prescription to children under 5 years of age?”*. The participant was asked additional questions to elaborate on when they do/do not prescribe antibiotics or if they have had good/bad experiences with this. At the end of the interview they were asked to describe antibiotic resistance. After the interviews, participants were given a token gift equivalent of 3 GBP, as reimbursement for their time.

### Data management and analysis

The interviews were recorded and transcribed verbatim by a member of the team (CM, EM, GN). In one interview the audio recording failed, in this case, the notes from the observing team member (GN) served as basis for analysis. Translation from Kiswahili to English were performed by clinicians at KCMC fluent in both languages. Part of the translated material from each translator was back-translated by FM to ensure quality and conformity of the translations. NVIVO 12 software (QSR International) was used for data management. Coding was performed by ME after data immersion. The emerging themes and categories were continuously tested against the data, through cycling between the categories and the transcripts [29]. The preliminary themes and categories were discussed and adapted by the co-authors FM, ME, ML, RA, RM and SS at regular seminars. A neutral co-examiner tested the results to ensure correct data analysis. These categories were presented to the co-examiner who assigned the uncoded quotations to the most appropriate category. Agreement was almost unanimous between the authors and the co-examiner.

### Ethics

The study was approved by the Kilimanjaro Christian Medical University College Research Ethics and Review Committee in Moshi, Tanzania (No. 2415) and the National Institute for Medical Research in Dar es Salaam (Vol. IX/3106). The Kilimanjaro Regional Medical Officer and the District Medical Officer of Health in Moshi Municipal and District Councils were informed of the study and gave their permission to visit the appointed health facilities. TAMISEMI, a Swahili acronym for the President's Office, Regional Administration and Local Government Tanzania (PO-RALG), were also informed of the study and provided written consent for its execution. All participants received both written and verbal information about the aims of the study and signed consent forms prior to the interviews. The potential power imbalance of a western, female researcher (ME) being part of the team conducting the interviews was taken into consideration. This was counteracted by the local researchers taking the leading role as the main interviewers and conducting the interviews in the participants’ native language, Kiswahili.

## Findings

**Fig. 1:**
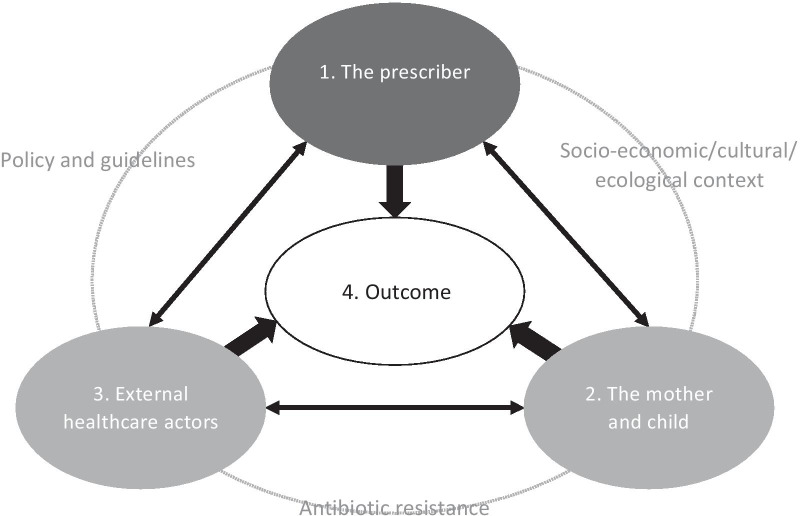
Relationship between the four themes of conceptions emerging in the analysis of in-depth interviews with primary health care workers on experiences of antibiotic prescription in children under the age of five, Moshi, Tanzania. Grey text refers to factors affecting antibiotic prescription and outcome that were evident to some, but not all, the participants

Four conceptual themes emerged during the analysis of the healthcare workers’ individual interview transcripts: (1) *Conceptions in relation to the prescriber,* (2) *Conceptions in relation to the mother and child,* (3) *Conceptions in relation to other healthcare actors,* and (4) *Conceptions in relation to outcome*, with associated categories (Table 2). An overview of how the themes are interrelated is shown in Fig. 1. Further, which themes and categories that appeared in each individual interview are found in Table S1 (Additional file 2).

**Table 2:** *Themes and categories that emerged in the analysis*

**1. Theme**	**Conceptions in relation to the prescriber**
1.1 Category	Executing clinical investigation
1.2 Category	Utilising structural support
1.3 Category	Treating what is not known
**2. Theme**	**Conceptions in relation to the mother and child**
2.1 Category	Antibiotic misuse is common practice
2.2 Category	Use of local remedies are less of a concern
2.3 Category	Low-income affects health care seeking behaviour and treatment
**3. Theme**	**Conceptions in relation to external healthcare actors**
3.1 Category	Health ministries and drug companies are accountable
3.2 Category	Pharmacies facilitate availability without prescription
3.3. Category	Some healthcare providers are dubious
**4. Theme**	**Conceptions in relation to outcome**
4.1 Category	Success is the norm
4.2 Category	Challenges are complex
4.3 Category	Antibiotic resistance is partly acknowledged

### 1. Theme: Conceptions in relation to the prescriber

The first theme contains conceptions related to the prescriber’s own internal process and how much room for manoeuvre there was when prescribing antibiotics for children under the age of five. The theme consists of the following three categories: *Executing clinical investigation*, *Utilising structural support* and *Treating what is not known*.

#### 1.1 Category: Executing clinical investigation

This category covers conceptions related to the healthcare workers’ basis for deciding when they do or do not prescribe antibiotics for children. The participants emphasised that a thorough physical examination and medical history provided by the mother was often sufficient to determine the need for antibiotics.

“When I get a patient coughing, I examine. Maybe he/she has a common cold or upper respiratory tract infection that are caused by viral infection, I don’t treat with antibiotics. Indications like productive cough with yellowish sputum, chest tightness and nasal flaring indicate that the child has severe pneumonia so this leads me to a conclusion that the patient needs antibiotics.” (Healthcare Worker (HW) 20)

In some statements, healthcare workers indicated that they used laboratory tests such as complete blood count, rapid diagnostic tests for malaria and urine dip-stick based on availability at the clinic or external laboratories. However, the clinician tended to lean towards their previous, clinical experience when the results were conflicting. *“(…) many times, bacterial infection does not depend on lab investigation. What I have taught myself is about the signs and the presentation of the mother.” (HW 16).*

The healthcare workers thus agreed that the physical examination and medical history of the child were their most important tool for antibiotic decision-making. The extent to which they used laboratory investigation varied according to availability but this was of secondary importance.

#### 1.2 Category: Utilising structural support

The second category comprises conceptions of the structural support the healthcare workers made use of that affected their prescribing habits. These include the use of guidelines, collegial support and continuous education. According to the participants, guidelines such as WHO’s Integrated Management of Childhood Illness (IMCI), are a trustworthy tool for deciding which antibiotics to choose for a certain condition or disease.

“Most of the times we use the WHO (…) Integrated Management of Childhood Illness in categorizing children diseases. So, from these guidelines we get the directives for prescribing antibiotics to children under 5 years.” (HW 1)

Statements from healthcare worker’s in larger facilities described a practice of consulting colleagues with higher education or discussing difficult cases at internal meetings. Continuous education and external seminars were valued by the participants for increasing knowledge and correcting malpractice, but also for creating a sense of belonging.

“Sometimes we prescribe underdoses as I mentioned in the first case (…) [therefore] we need to be people who read a lot/keep updated. For example, every Thursday there are meetings at [a referral hospital] where they invite people from different centres where they share/teach so you learn, you get exposure and feel you belong to each other, which is very useful.” (HW 8)

Some healthcare workers requested more training on antibiotic resistance and others stated that previous training has, in fact, affected prescribing practices. *“Previously we used to give medicine to children without investigations. But after I went for further studies, I realized that not all children are to be given antibiotics.” (HW 15)*.

In summary, guidelines such as the IMCI are a trusted tool for antibiotic prescription for children and the healthcare workers had a positive view of collegial, continuous education as a means of gaining support and new knowledge.

#### 1.3 Category: Treating what is not known

Statements in the third category revealed a variation in terms of how the healthcare workers have dealt with uncertainty when diagnosing and treating children. Some statements highlight that clinicians were comfortable with giving advice for symptomatic treatment after having ruled out severe disease or specific diagnoses requiring antimicrobial treatment. *“One child presented with flu and high fever, but on examination for chest tightness or drowsiness I found no problem. I advised the mother to give black tea and lemon and the child recovered.” (HW 1)* Other healthcare workers tended to prescribe antibiotics for children with fever without focus and/or negative laboratory results on the basis of their previous experience, as shown in the following statement:

“Other times I give antibiotics even if there are no positive results from the lab investigations, since the child had other signs/symptoms of other diseases. I treat in this way because I cannot send home a child who comes to me with high fever without giving any treatment.” (HW 10)

At times, the primary health clinicians have come across children who they perceived as being severely ill, requiring urgent management. In this case, statements show that children may be given antibiotic treatment before the diagnoses are being established, and some are subsequently admitted or referred to a higher-level facility.

“(…) for example, if the child comes with high fever and convulsions you cannot know if it is malaria or septicaemia, so here, I will give a combination of drugs or antibiotics while waiting for the test results for malaria.” (HW 16)

When dealing with uncertain cases of childhood infections, perceived severity of illness is an important factor when determining the need for antibiotics. However, for children without apparent need for antibiotics, statements indicate that the healthcare workers had different strategies depending on their self-confidence in giving advice for non-antibiotic treatment.

### 2. Theme: Conceptions in relation to the mother and child

The second theme comprises conceptions of the social and relational aspects of the mother and child. This theme consists of the following three categories, namely *Antibiotic misuse is common practice*, *Use of local remedies are less of a concern* and *Low-income affects healthcare seeking behaviour and treatment*.

#### 2.1 Category: Antibiotic misuse is common practice

Statements in this category revealed that healthcare workers frequently come across misuse of antibiotics in children. Many healthcare workers are approached by mothers expecting a prescription for antibiotics for their child or have encountered children in whom antibiotic treatment had been initiated at home. Non-compliance to treatment, such as not finishing the course of treatment or not adhering to time intervals for administration, was said to be common.

“In fact, the main challenge is the children not completing the doses. The parent can be instructed to give the dose for 5 days but they only give it for 3 days when they see improvements to the child health, but after a certain period the child suffers the very same problem.” (HW 19)

The statements show how the healthcare workers have handled these challenges, which for some have been a cause of frustration.

“(…) there are challenges. For example, a mother can tell you that ‘my child is [not] cured without an injection’. Based on my experience, other times the child does not need medication. But when I try to explain it is a challenge! Others may think you don’t like them (Humpendi), or I am a stingy person (Mchoyo) in [not] giving the medication, but we prescribe medicine based on the guidelines!” (HW 3)

However, most clinicians emphasised the need for good consultation to promote appropriate antibiotic use, as the mothers may otherwise not adhere to the treatment, re-attend or buy antibiotics directly from the pharmacy.

“For instance, a parent can request you to prescribe Septrin as he/she believes it can cure the child. Mostly we prescribe medications after doing a thorough clinical assessment, investigations and proper counselling to the mother after establishing the diagnosis. Most parents tend to understand and are satisfied when they are counselled well.” (HW 19)

In conclusion, the statements show that misuse of antibiotics and expectations of antibiotic prescription for Tanzanian children constitute a common challenge to primary healthcare workers. Many expressed the need for educating the mothers on rational antibiotic use, meanwhile, some have tried to execute this time- and energy-consuming task in their daily, clinical practice.

#### 2.2 Category: Use of local remedies are less of a concern

While not present in all the interviews, statements reveal that the healthcare workers have sometimes come across mothers who had used local remedies, such as herbal medication or bicarbonate soda, in the children. This was generally less of a concern for them:

“If they [the mothers] have used traditional medicine (dawa) and they didn’t give the child infection or any reaction (…) I will give this child a dose of medication (…). Because traditional herbal medicines (mitishamba) are only leaves and I don’t think they affect the child negatively.” (HW 16)

Thus, some did not perceive any negative effects of traditional, herbal medicine, whilst others have been discouraging the use of these: *“There are some who tell us they are using local herbal medications (mitishamba), and we discourage the use of these because they do not have any standards (hazina viwango vyovyote).” (HW 20)*.

#### 2.3 Category: Low-income affects healthcare seeking behaviour and treatment

According to the statements in the third category, the social status of the mother and child affected healthcare seeking behaviour and, consequently, the use of antibiotics. Some healthcare workers at private facilities recognised that low-income families, due to financial constraints, were more likely to buy antibiotics for their children straight from the pharmacy, without first attending the health facility:

“(…) when a low-income family comes to the health facility (hospitali) there are charges like consultation fee, laboratory investigations fee. So, they skip that and go to the pharmacies and explain what is their problem and they buy [antibiotics].” (HW 8)

Further, according to the statements lack of money may also force families attending private healthcare to buy only a portion of the recommended course of antibiotics. It was obvious that few healthcare workers at public facilities had also taken the financial situation into account when managing a child in need of antibiotics. *“For instance, in patients with financial constraints we normally prescribe on the basis of what is available in the facility.” (HW 6)* Children from low-income families may thus more often have been treated with antibiotics chosen on the basis of availability or cost, rather than what was most eligible for the condition.

In some of the statements it was mentioned that financial constraints may also present a barrier to the use of laboratory tests, as these were often performed at external laboratories. *“Sometimes there are complications and we ask them to have laboratory investigations done. Those who can afford to pay goes for that.” (HW 3)* According to the data, social status is thus one of the determinants of antibiotic use in Tanzanian children due to occurrent costs or resource limitations at the health facility, laboratory or pharmacy.

### 3. Theme: Conceptions in relation to other healthcare actors

The third theme deals with conceptions in relation to other healthcare actors, namely, how they affect the quality, availability and use of antibiotics. The three categories are: *Health ministries and drug companies are accountable*, *Pharmacies facilitate availability without prescription* and *Some healthcare providers are dubious*.

#### 3.1 Category: Health ministries and drug companies are accountable

This category covers conceptions of health ministries and drug companies’ responsibility in providing adequate antibiotics in terms of quality, range and quantity. At first, statements in this category revealed a concern about the quality of the available antibiotics:

“To the drugs manufacturing companies, I say that they have to consider the issue of humanity! They have to produce medicines that have qualities. Most of the medicine are of a very low quality/standard. They don’t treat as we expect.” (HW 12)

Thus, the quality of drugs was perceived as the liability of the manufacturing companies and this should be dealt with by the Tanzanian authorities, according to the following statement:

“The government should make a policy [of control]. One particular medicine that enters the country today and one that will be imported after one year look the same, but they have different qualities.” (HW 17)

Secondly, some healthcare workers in public facilities experienced problems with receiving an adequate range and quantity of antibiotics from the governmental Medical Stores Department. This limited their choices in terms of antibiotic prescription which, according to some participants, may lead to antibiotic resistance. *“Another major problem that can cause antibiotic resistance is limited antibiotic stock. We need an adequate stock in our facility.” (HW 6)*.

In summary, some healthcare workers were concerned that certain antibiotics available in Tanzania were of poor quality and called for increased control by the health ministry. According to the statements, limited range of antibiotics may affect antibiotic resistance.

#### 3.2 Category: Pharmacies facilitate availability without prescription

The second category contains conceptions of the relationship between the healthcare worker and the pharmacies. According to the statements, pharmacies frequently sell antibiotics without a prescription, which may lead to irrational use of the antibiotics.

“We have parents like this, they (…) went to pharmacy and were given medicine but there was no improvement to the child. I have a look at the medicine, I assess the child to rule out what the problem is, and we found that the child was receiving wrong treatment! (…) I advise them not to start in the pharmacies when the child is sick. They have to come to the hospital; I prescribe, then they can go and buy in the pharmacies.” (HW 20)

On the contrary, few statements recognized prescription by pharmacists as a positive means of decreasing the work load of the health facilities. *“The stores [pharmacies] are so useful that we were not able to do all tasks alone in the health facilities. I advise if people that, if see the problem is big, they have to go to the health facility (hospitali).” (HW 8)*.

Nevertheless, most healthcare workers perceived the common practice of pharmacies in selling antibiotics without a prescription as problematic, leading to irrational use of antibiotics in Tanzanian children.

#### 3.3 Category: Some healthcare providers are dubious

The third category covers statements relating to the healthcare workers’ concerns about the management and treatment of children at other clinics. Some less well-equipped or rural facilities are perceived to be lacking in diagnostic accuracy.

“(…) many times there is a problem in the dispensaries and rural hospitals and many times they do not get to the investigation. They rush to give antibiotics to the children trying to save them but the child guidelines [IMCI] will help to know if the child should be given antibiotics or should be tested further.” (HW 16)

Private facilities, on the other hand, may be driven by profit as shown in the following statement:

“I have worked in many private clinics where the patients come and buy half a dose, and I tell them that it can bring problems to them. For example, they want to buy only 15 amoxicillin tabs [half a dose], but because our boss wants income we have to give the medication.” (HW 3)

Notably, both these two examples of perceived dubious management may lead to the irrational use of antibiotics, according to the participants.

### 4. Theme: Conceptions in relation to outcome

The fourth conceptual theme involves conceptions of treatment outcome when prescribing or choosing not to prescribe antibiotics to children. The theme consists of three categories: S*uccess is the norm, Challenges are complex* and *Antibiotic resistance is partly acknowledged*.

#### 4.1 Category: Success is the norm

Statements in this category revealed that, overall the healthcare workers had positive experiences when treating children with antibiotics. Some also had positive experiences of when they chose to not prescribe antibiotics in cases of perceived dehydration fever in neonates, teething or common colds.

“I have met many children with upper respiratory infection and I give antibiotics based on the guidelines, and I have never met a child coming back to me with the same problem. I have experience of not giving antibiotics to neonates for a long time. Most of the time neonates suffer dehydration fever and I encourage the mothers to breastfeed and they recover.” (HW 1)

Several statements showed that mothers were instructed to come back to the clinic if the child did not improve. If the mother did not return, the healthcare workers interpreted this as a sign of treatment success. *“Once we prescribe medication, we tell them to come back if the condition worsens. Many don’t come back and this gives us a feedback of a positive effect of the medicine given.” (HW 3)*.

In general, children treated with or without antibiotics after assessment by the primary healthcare workers recovered well. If they visited the clinic again this was mainly due to a new condition or disease, according to the statements.

#### 4.2 Category: Challenges are complex

Participants identified a wide variety of challenges perceived to have an adverse effect on treatment outcome. These statements included non-compliance to treatment, self-prescription, misdiagnosing, drug resistance and lack of resources. In response to this, the healthcare worker may have changed treatment or referred the child to a higher-level facility.

“This [treatment failure] happens in relation to [work] resources (…) As a doctor one has to fight in all ways possible to do the job because there are times drugs become resistant or there is a misdiagnosis and you have to look for an alternative or make a referral if the patient doesn’t get well; they will be required to go to a higher-level hospital; you cannot do everything right.” (HW 16)

A common challenge for the healthcare workers was the lack of adherence to antibiotic administration in children treated at home, which according to some was a reason for treatment failure in the children. Others attributed disease re-occurrence to the effects of the cold season. *“At our centre there comes situations where the child comes with the same problem and this is mainly noted during dusty or cold seasons when children cough a lot.” (HW 10)*.

In summary, the challenges facing primary healthcare workers in Tanzania when prescribing antibiotics to children were complex according to the statements. There were multiple causes of adverse outcomes including non-compliance with treatment.

#### 4.3 Category: Antibiotic resistance is partly acknowledged

This category comprises conceptions of antibiotic resistance, including statements relating to perceived mechanisms of antibiotic resistance and to whom this conveyed a challenge. Most healthcare workers were aware of the issue of antibiotic resistance, but few experienced it as a problem in daily practice. *“I have never come across that [antibiotic resistance]. We are in the primary level [in the healthcare system] so it is very difficult to notice that.” (HW 1)*.

Whilst some statements showed no conception of the mechanism of antibiotic resistance, the remaining statements portrayed three main explanations for the development of resistance. The first was related to ‘the pathogen’: *“This [antibiotic resistance] is when a pathogen or bacteria adapts to a certain medicine, when given in small amount that cannot kill it. When the pathogen/bacteria rise up again strongly and the medicine cannot help/treat anymore you have to do loading or change the medicine.” (HW 3)*.

The second conception was related to ‘the disease’: *“Drug resistance is when a patient is given medicine and gets cured, and when he is ill again, he is treated with the same medicine and finally the disease becomes resistant to that medicine.” (HW 7)*.

The third conception concerned the mechanism of antibiotic resistance related to ‘the body’: *“The way I understand [antibiotic resistance], is when a person uses many medicines to treat one condition. So, when a drug is used it can no longer treat because the body gets used to the drug.” (HW 4)* However, a common thread between these three conceptions was that individual misuse of antibiotics could drive the development of resistance and consequent treatment failure in that same individual. Meanwhile, few healthcare workers reflected on how misinterpretation of the IMCI guidelines has affected prescription of amoxicillin and thus may contribute to resistance as shown in the following statement:

“This [antibiotic resistance] can come about when the medication is used irrationally or if the same drug is used for a long time (…) for example these drugs, amoxicillin, came in 1999 in the training of the IMCI. It was one of the recommended drugs and thus was used a lot [literally] every patient who came was given this drug to make them happy but right now this drug does not work well.” (HW 16)

In summary, most healthcare workers perceived antibiotic resistance as a problem for the individual who has been misusing antibiotics. However, few reflected on the issue in relation to its effect on public health.

## Discussion

The issue of antibiotic resistance is multifaceted and highly affected by human behaviour at different levels [30]. Prescribing clinicians in primary healthcare play a key role in the use of antibiotics in the community, by providing prescriptions, health education and setting standards concerning when antibiotics may be used. This can be mirrored in possible self-mediciation [16]. Our study highlights the complexity of clinical decision-making regarding appropriate antibiotic use in Tanzanian children, and describes how primary healthcare workers perceive and confront these challenges. Understanding healthcare workers’ conceptions of antibiotic prescription and antibiotic resistance is central to efficiently promoting antibiotic stewardship in the local context [31].

Four main themes emerged in the analysis. The first theme *“Conceptions in relation to the prescriber”*, contains rich material in which the most important finding was the agreed importance of physical examination and history from the mother as the main basis for deciding on antibiotic or non-antibiotic treatment for children, aligning with the IMCI guidelines. The variation was mainly in how confident the clinicians were in giving advice for non-antibiotic treatment. The second theme *“Conceptions in relation to the mother and child”*, revealed how common it was for the clinicians to come across misuse and self-prescription of antibiotics in children whilst the use of local remedies was less of a concern for them. It further reveals that social status may be a determinant for antibiotic (mis)use in Tanzanian children. The third theme *“Conceptions in relation to external healthcare actors”*, contains the healthcare workers’ concerns regarding the quality of available antibiotics and examples of irrational antibiotic use caused by pharmacies and/or dubious healthcare providers. The fourth theme *“Conceptions in relation to outcome”*, showed that although antibiotic resistance was perceived as a problem for the individual in which antibiotics are used irrationally, this was generally not a concern for the clinicians on a day-to-day basis or as an emerging threat for effective treatment of bacterial infections in the community. Based on these results we analyse and discuss implications of our findings, which are of importance for future policy-making or research.

Previous studies show IMCI training improves quality of care and antimicrobial prescription by Tanzanian healthcare workers [32, 33]. However, adherence rates to the IMCI guidelines have been low [34] with a significant gap between actual knowledge and implementation thereof [35]. In our study, the participating healthcare workers express trust in the IMCI [36], but the extent to which they follow the guidelines was not explored. Statements reveal that many healthcare workers had met mothers requiring an antibiotic for their child or who had initiated antibiotic treatment without prescription. Responding to this was a common time- and energy-consuming challenge for the clinicians. Most, but not all healthcare workers felt comfortable providing advice for symptomatic treatment in febrile children once need for antibiotics or anti-malarias had been ‘ruled-out’, although time was a limiting factor. Previous studies in high income settings show that parents are more likely to accept non-antibiotic treatment if they are presented with a specific recommendation of ways to help the child to feel better [37, 38]. Current IMCI guidelines are less supportive for clinicians in terms of providing instructions for symptomatic treatment, whilst focusing on recognizing the severely ill and treating few conditions such as bacterial pneumonia, malaria, HIV and malnutrition [39]. Recent studies in Tanzania suggest that most cases of acute febrile illness in children are in fact, similar to high-income settings, due to viral pathogens whilst serious bacterial illness is likely to be decreasing due to vaccine implementation and improved hygiene [40–42]. New, or updated, guidelines and training should include more specific instructions on supportive care which will enable both healthcare workers and parents to refrain from the unnecessary use of antibiotics in common viral illness whilst attending to the overall well-being of the ill child [43]. This may also increase mothers’ trust in the healthcare workers’ patient management and prevent mothers from continued, costly care-seeking at private healthcare providers or pharmacies.

Furthermore, our study demonstrates that some primary care facilities in Moshi make use of laboratory tools such as complete blood count or urinary dip-stick for febrile children. However, the interpretation and consequence of each of these laboratory tests were not explored, but the healthcare workers generally emphasized that the clinical examination was of prior importance. Other statements indicated that laboratory tests would entail an additional cost, not always possible for the mother to afford. Current IMCI guidelines include only laboratory tests for malaria whilst novel algorithms such as the ALMANACH have included urinary dip-sticks [44] and the e-POCT which make use of additional point-of-care tests including oxygen saturation, haemoglobin and inflammatory markers (C-reactive protein (CRP) and procalcitonin) [45]. The latter algorithm was tested in an RCT on febrile children attending outpatient clinics in Dar es Salaam, showing promising results in improving clinical outcome whilst also decreasing unnecessary antibiotic use. On the other hand, a study from Muheza district, Tanzania performing point-of-care tests (CRP and white blood cells) on malaria negative, febrile children concluded that assessment of white blood cell counts had limited value for detecting bacterial disease. In addition, using CRP measurements alone as a predictor for antibiotic treatment was inadequate [46]. This highlights that to benefit the ill child and guide clinicians on antibiotic prescription, point-of-care tests such as CRP and procalcitonin need to be introduced alongside updated guidelines to assist with interpreting the results and the clinical findings. Significantly, our study shows that there are several barriers to a successful implementation of point-of-care tests in Tanzania. Firstly, primary healthcare workers are accustomed to relying only on their clinical examination and medical history from the mother. Thus if not implemented alongside sufficient training, the use of point-of-care tests may change little in their antibiotic prescribing behaviour. Secondly, if not implemented free of charge, financial constraints may be a major barrier for the use of point-of-care tests in the general child population.

Our study suggests few possible mechanisms that could link enhanced antibiotic resistance to low socioeconomic status. According to the healthcare workers in our study, despite the policy of free healthcare for children under-five, low-income families were more often buying antibiotics for their children straight from the pharmacies, without letting the child be seen by a clinician because of the occurring costs. Further, low-income families could less often afford laboratory tests and would more often be prescribed antibiotics based on availability. This suggests more frequent irrational use of antibiotics in children from low-income families and need for improvement in the healthcare system to achieve equitable healthcare for children in Tanzania. Also, poor sanitary standard and use of antibiotics in animals and agriculture contributes to antibiotic resistance in the community [11] with children of low-income families being more exposed to a contaminated environment. Future studies should explore associations between antibiotic resistance and socio-economic status in low- and middle-income countries to better understand possible mechanisms and which interventions would most efficiently protect vulnerable groups from resistant pathogens.

Healthcare workers in our study had different conceptions of antibiotic resistance in terms of mechanism, i.e. if it is related to the pathogen or bacteria, which is correct, or if it is related to the body, which is incorrect. However, they all viewed antibiotic resistance as a concern for the individual consuming the antibiotics. There is indeed an association between antibiotic consumption and the subsequent development of resistance on an individual level, as in the case of antiretroviral treatment. But, perhaps of greater importance when it comes to antibiotic resistance is the association between high use of antibiotics and increased antibiotic resistance at the community, country and regional levels [47]. Previous studies in high-income settings show clinicians who are aware that antibiotic resistance is a serious threat, reflecting on the consequences in broader terms rather than in the individual patient, are more likely to practice antibiotic stewardship [21, 23]. Perceived risks in the individual patient have proved to be a major barrier for more conservative prescribing as the risk of increased antibiotic resistance may, on the other hand, seem more abstract or remote [48]. Further, surveillance of the susceptibility of human pathogens is generally deficient in low-income countries, although it is an important tool in adjusting treatment guidelines and justifying continued efforts on rational antibiotic use [49]. In conclusion, preserving antibiotics for the future demands a ‘One Health’ approach [12], including comprehensive efforts to raise the standard of sanitation, providing clean water, implementing surveillance and building awareness of the effects of today’s antibiotic usage for the health of humanity tomorrow.

The interviewed healthcare workers were suspicious as to whether some antibiotics on the market met the required quality standards and attributed experienced treatment failure to low-quality drugs. However, it is difficult to determine without further investigation whether these experiences may, in fact be due to other causes such as mis-diagnosing, antibiotic resistance or failed adherence to treatment. Nevertheless, these experiences are in line with the growing evidence that falsified and substandard medicines are increasing as a worldwide problem [50]. This disproportionally affects those low- and middle-income countries that have weaker pharmaceutical regulations, less advanced technical capacity and poor supply chain management [51, 52]. A review published in 2018 estimated the overall prevalence of poor-quality medicines in Africa to be as high as 19% [53]. This is causing huge economic losses and an estimated 150,000–400,000 unnecessary child deaths due to malaria and childhood pneumonia [52]. A study on the cost of confiscated falsified and substandard medicines and cosmetics by the Tanzanian authorities between 2005 and 2015 showed an increase in quantity and cost of these products over time, which may in part reflect increased regulatory capacity [54]. For antimicrobials, falsified or substandard antimalarials have been mostly studied [55–57]. A recent study conducted in Nairobi county assessed the quality of amoxicillin and amoxicillin/clavulanic acid on the market. This indicated that 38% of the samples, all of which were imports, failed to meet the standards of the United States Pharmacopoeia [58]. This was mostly due to failing uniformity of weight for amoxicillin tablets or the chemical content for amoxicillin being too low in the oral suspensions at day seven. It is important to note that the antibiotic suspensions were being kept in room temperature to mirror the fact that the majority of households in low-income countries do not have access to refrigerators, where the suspensions are supposed to be stored. A study from Tanzania, including Kilimanjaro region, testing the effectiveness of purchased antibiotics against reference bacteria, also suggested the presence of some substandard batches although no falsified antibiotics were found [59]. The consumption of substandard antibiotics is of concern since it endangers not only the management of associated bacterial infections, but also enhances antibiotic selection pressure leading to increased antibiotic resistance and consequent use of more expensive, broad-spectrum antibiotics [3]. Furthermore, although the majority of antibiotics on the market are seemingly of sufficient quality, the erosion of trust in the industry and health ministries has had a detrimental effect. This may, in turn decrease adherence to the treatment options recommended by the authorities. In summary, strengthening pharmaceutical governance on several levels such as post-market quality control of antibiotics and legislative enforcement of prescription-only dispensing, is important in order to promote trust between healthcare workers and other stake-holders, this being a prerequisite for adherence by clinicians to any effort towards achieving increased antibiotic stewardship.

Several topics were raised in the interviews that warrant further research. First, few healthcare workers in our study mentioned meeting children who had been given local remedies, among them herbal medication, prior to their visit. Although it was discouraged by some, others did not perceive any adverse effects. Use of traditional or herbal medication is known to be common across Sub-Saharan Africa and in Tanzania [60, 61]. However, a previous study from Moshi revealed that mothers preferred biomedical medicine for their children whilst traditional medicine was perceived as belonging to the past [62]. Whilst some herbs may indeed be harmless, there are several reasons to be cautious about the use of herbal medication in children as, for example, they share the same drug metabolising enzymes and drug transporters as prescribed drugs, which substitute a risk for adverse interactions [63]. Further, the low body weight of children makes them more sensitive to over-dosing of compounds which may not be harmful for adults. Also, the use of local remedies in children may cause a delay in seeking appropriate healthcare [61]. Future studies should explore current use of local remedies in Tanzanian children in both urban and rural areas, aiming to discern what may be endorsed or discouraged by the health ministries. Healthcare workers need to be educated in routinely raising the potentially sensitive topic of local remedies with a non-judgmental approach, for mothers to listen to their careful and specific advice [60].

Finally, to our surprise, three healthcare workers (HW 1, 5 and 18) reported positive experiences of not giving antibiotic treatment to small babies presenting with fever, on the notion that lack of feeding caused the temperature to rise. This was in contrast to the IMCI guidelines which classifies all infants up to 2 months with high or low temperature (≥ 37.5 °C or < 35.5 °C) as being eligible for intramuscular antibiotic treatment and, if possible, referral to a higher-level facility [39]. Because serious bacterial infections are associated with high morbidity and mortality in neonates, vigilant approaches to treatment have been established. However, the association between fever and clinical and laboratory signs of dehydration in otherwise healthy, term infants has recently been described by retrospective, single-centre studies from Israel, Canada and the US, [64–66]. These studies included febrile newborns after 24 h but during the first week of life and suggest dehydration to be the most common cause of fever in this group, but significantly, the nature of these studies made it impossible to determine a causal relationship between dehydration and fever in the selected cases. Infants born in low-income countries are at higher risk of infection, but may also be prone to dehydration due to higher environmental temperature generally and low use of postnatal care [67]. Future, prospective multi-centre studies in middle- and low-income countries are needed to determine in which cases of neonatal fever it is considered safe to refrain from antibiotic treatment in favour of cooling or fluid therapy alone.

### Strengths and limitations

The present study exhibits both strengths and limitations. In total, 20 primary healthcare workers were included. This is in line with the 15–20 participants considered to be a sufficient number to reach the variability in experiences warranted in phenomenography. The majority of the included healthcare workers (16/20) worked in an urban or sub-urban area, the variability of the data is therefore limited in this aspect. However, a wider variety is involved in terms of participants’ educational backgrounds, age and years of experience. The first author of this study (ME) is not Kiswahili speaking and thus did not conduct interviews but observed a portion of the interviews and took part in the debriefing. Efforts were made in counteracting a possible power-imbalance by the presence of a western, female researcher. In retrospect, this did not appear to affect the openness of the interviews she was observing, perhaps primarily due to the high social status held by the healthcare workers in Tanzania. During analysis of the transcribed interviews, close contact was maintained between the first and second author (RM) to discuss the English translation of the transcripts and cultural aspects.

This study describes the experiences of healthcare workers in Northern Tanzania, a relatively wealthy part of the country where the majority of the inhabitants live reasonably close to a health clinic. Most participants worked in an urban area where malaria transmission was low. Healthcare workers serving in similar contexts, in other parts of East Africa may share similar experiences, but the results should be transferred with caution.

## Conclusions

Through in-depth interviews with primary healthcare workers prescribing antibiotics to children in Northern Tanzania, our study provides insights which are essential for shaping effective interventions accelerating antibiotic stewardship within the community. To increase rational antibiotic prescription, awareness need to be raised among Tanzanian primary healthcare workers of the threat of antibiotic resistance, not only to a few individuals, but also to public health. Further, there is a need for improved pharmaceutical governance, including legislative enforcement of prescription-only dispensing and post-market quality control of antibiotics, to ensure trust and accountability between the different healthcare actors. Guidelines on childhood illness should be updated with information and advice concerning symptomatic treatment when antibiotics are not necessary to support rational prescribing practices and to promote trust in the clinician-mother relationship.

## Supplementary Information

**Additional file 1.** Interview guide (in English and Kiswahili).

**Additional file 2: Table S1.** Themes and categories that emerged in the analysis in relation to the individual participants.

## Data Availability

The datasets generated and analysed during the current study are not available due to confidentiality clauses but anonymised versions are available from the corresponding author upon reasonable request.

## Abbreviations

HW: Healthcare worker
IMCI: Integrated Management of Childhood Illness
KCMC: Kilimanjaro Christian Medical Centre
WHO: World Health Organisation
