# Supplementary material for: Tanzanian primary healthcare workers’ experiences of antibiotic prescription and understanding of antibiotic resistance in common childhood infections: a qualitative phenomenographic study
Source: Antimicrob Resist Infect Control. 2021 Jun 27;10:94. doi: 10.1186/s13756-021-00952-5 (PMC8237496; doi:10.1186/s13756-021-00952-5)
Supplement: Supplementary file 1 — Additional file 1. Interview guide (in English and Kiswahili). [file 13756_2021_952_MOESM1_ESM.docx]

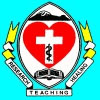

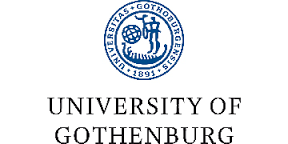


**Interview guide for prescribing Healthcare Workers**

1. **Screening question**

**Do you have experiences of prescribing antibiotics to children under 5 years of age?**

1. **Opening question**

Can you please describe your experiences of antibiotic prescription in children under 5 years of age?

1. **Follow up questions**
   1. How do you decide when to prescribe an antibiotic to a child under 5 years of age?
      1. What specific factors influence if you prescribe an antibiotic to a child?
      2. What factors influence if you **do not** prescribe an antibiotic to a child?
      3. Can you describe how you examine and treat a child that presents at the clinic with fever and respiratory tract symptoms?
   2. Have you had **any good** experiences when choosing to prescribe or not prescribe an antibiotic to a child? Can you please give an example?
   3. Have you had **any difficult** experiences when choosing to prescribe or not prescribe an antibiotic to a child? Can you please give an example?
   4. Have you ever experienced prescribing an antibiotic to a child, but the child did not improve? Can you please give examples? How did you manage?
   5. Can you please describe antibiotic resistance?
      1. Is this of concern to you? Why? Why not?
      2. Do you have any suggestions of what can be done to not increase antibiotic resistance?


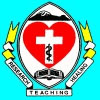

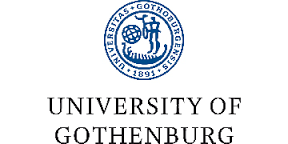


**Interview guide for prescribing Healthcare Workers**

**1. Maswali ya awali**

**Je una uzoefu wowote wa utoaji wa dawa za antibiotiki kwa watoto chini ya umri wa miaka mitano?**

**2. Utangulizi**

Je unaweza kunielezea uzoefu wako kwenye kuota dawa za antibiotiki kwa watoto chini ya miaka mitano?

**3. Maswali ya kufuatilizia**

3.1 Nielezee ni wakati gani unafikia kufanya maamuzi ya utoaji dawa za antibiotiki kwa watoto chini ya miaka mitano?

3.1.1 Ni nini haswa kinakupelekea kutoa antibiotiki kwa mtoto ?

3.1.2 Nini inakupelea kutokutoa antibiotiki kwa mtoto?

3.2 Katika uzoefu wako wa kutoa antibiotiki unaweza kunielezea matokeo chanya ambayo umekutana nayo katika utoaji au kutokutoa antibiotiki? Una mfano?

3.3 Katika uzoefu wako wa kutoa antibiotiki unaweza kunielezea matokeo changamoto ambayo umekutana nayo katika utoaji au kutokutoa antibiotiki? Una mfano?

3.4 Je katika uzoefu wako ulishawahi kutoa dawa za antibiotiki kwa mtoto lakini hakupata nafuu yoyote? Una mfano wowote? Ulichukuwa maamuzi gani?

3.5 Unaweza kunieleza usugu wa antibiotiki ni nini?

3.5.1 Je hili ni tatizo? Kwanini ndio/hapana?

3.5.2 Je una mawazo gani kushusu nini lifanyike kuhusu kutoongezeka kwa usugu wa dawa za antibiotiki?
