# Supplementary material for: Tanzanian primary healthcare workers’ experiences of antibiotic prescription and understanding of antibiotic resistance in common childhood infections: a qualitative phenomenographic study
Source: Antimicrob Resist Infect Control. 2021 Jun 27;10:94. doi: 10.1186/s13756-021-00952-5 (PMC8237496; doi:10.1186/s13756-021-00952-5)
Supplement: Supplementary file 2 — Additional file 2: Table S1. Themes and categories that emerged in the analysis in relation to the individual participants. [file 13756_2021_952_MOESM2_ESM.docx]

## **Additional file 2.**

**Table S1**. Themes and categories that emerged in the analysis in relation to the individual participants.

| **Healthcare Worker** | **1** | **2** | **3** | **4** | **5** | **6** | **7** | **8** | **9** | **10** | **11** | **12** | **13** | **14** | **15** | **16** | **17** | **18** | **19** | **20** | **Total** |
| --- | --- | --- | --- | --- | --- | --- | --- | --- | --- | --- | --- | --- | --- | --- | --- | --- | --- | --- | --- | --- | --- |
| **Theme1. Conceptions in relation to the prescriber** |  |  |  |  |  |  |  |  |  |  |  |  |  |  |  |  |  |  |  |  | **20** |
| 1.1 Executing clinical investigation | x | x | x | x | x | x | x | x | x | x | x | x | x | x | x | x | x | x | x | x | 20 |
| 1.2 Utilising structural support | x | x | x | x | x |  |  | x | x |  | x | x |  |  | x | x |  | x | x |  | 13 |
| 1.3 Treating what is not known | x | x | x |  | x | x | x | x | x | x | x | x | x |  | x | x |  | x | x | x | 17 |
| **Theme 2. Conceptions in relation to the mother and child** |  |  |  |  |  |  |  |  |  |  |  |  |  |  |  |  |  |  |  |  | **18** |
| 2.1 Antibiotic misuse is common practice | x | x | x | x | x | x | x | x | x | x | x |  | x | x | x |  |  | x | x | x | 17 |
| 2.2 Use of local remedies are less of a concern |  |  |  |  |  |  |  |  |  | x |  |  |  |  |  | x |  |  |  | x | 3 |
| 2.3 Low-income affects health care seeking behaviour and treatment |  |  | x | x |  | x |  | x |  |  |  |  |  |  |  |  |  |  | x |  | 5 |
| **Theme 3. Conceptions in relation to external health care actors** |  |  |  |  |  |  |  |  |  |  |  |  |  |  |  |  |  |  |  |  | **16** |
| 3.1 Health ministries and drug companies are accountable |  |  |  |  |  | x | x | x | x |  | x | x |  |  |  |  | x | x |  |  | 8 |
| 3.2 Pharmacies facilitate availability without prescription |  |  |  |  |  | x | x | x | x | x |  |  | x | x | x |  | x | x | x | x | 12 |
| 3.3 Some health care providers are dubious |  |  | x |  |  | x |  |  |  |  |  |  |  |  |  | x | x | x |  |  | 5 |
| **Theme 4. Conceptions in relation to treatment outcome** |  |  |  |  |  |  |  |  |  |  |  |  |  |  |  |  |  |  |  |  | **20** |
| 4.1 Success is the norm | x | x | x | x | x | x |  | x |  | x | x | x |  | x | x | x | x | x | x | x | 17 |
| 4.2 Challenges are complex | x | x | x |  | x | x | x | x | x | x |  | x | x |  | x | x | x | x | x | x | 17 |
| 4.3 Antibiotic resistance is partly acknowledged | x | x | x | x | x | x | x |  | x | x | x | x | x | x | x | x | x | x | x | x | 19 |
